# Management of Triple M Syndrome: A Systematic Review with Narrative Synthesis of Immune Checkpoint Inhibitor-Induced Myasthenia Gravis, Myositis and Myocarditis

**DOI:** 10.3390/cancers17132063

**Published:** 2025-06-20

**Authors:** Martin Furlepa, Isabella Watts, Aisling S. Carr

**Affiliations:** 1 Department of Neurology, University College Hospital, London NW1 2BU, UK; 2 Department of Oncology, Imperial College Healthcare NHS Trust, London W6 8RF, UK; 3 Centre for Neuromuscular Diseases, National Hospital for Neurology and Neurosurgery, UCLH, London WC1N 3BG, UK; 4 Department of Neuromuscular Diseases, Institute of Neurology UCL, London WC1N 3BG, UK

**Keywords:** immunotherapy, Triple M syndrome, overlap syndrome, immunotherapy toxicity

## Abstract

**Background:** The advent of immunotherapy has revolutionised cancer treatment by harnessing the immune system to target tumour cells. However, there is increasing awareness of immunotherapy-related adverse events, which can be severe and even fatal. While system-specific immune-related adverse events (ir-AEs) are well documented, growing evidence suggests the existence of overlap syndromes—distinct clusters of immune-mediated complications. One such syndrome is the overlap of myasthenia gravis, myositis and myocarditis, collectively known as Triple M (3M) syndrome. This syndrome is complex, varying in presentation and severity, with in-hospital mortality rates approaching 40%. Whilst there is consensus on the management of system-specific complications, there is no consensus guidance for the management of these overlap syndromes. **Methods:** In this paper, we conduct a review of the literature, analysing reported cases of 3M syndrome, focusing on treatment approaches and patient outcomes at an individual level. **Conclusions:** This review highlights the complexity of diagnosing and managing 3M syndrome due to inconsistent reporting, lack of standardised criteria for diagnosis, and treatment variability. While evidence remains limited, we offer broad clinical recommendations and underscore the urgent need for consensus definitions, prospective data collection, and structured treatment guidance.

## Simple Summary

Immunotherapy has transformed cancer treatment, but it can have serious side effects. One rare but dangerous complication is a combination of nervous system and muscle inflammation called Triple M (3M) syndrome, which includes myasthenia gravis (a neurological disorder causing weakness), myositis (muscle inflammation) and myocarditis (heart inflammation). This combination was not seen in clinical practice prior to the introduction of immunotherapy, so there has been much interest in how, and in whom, it presents, with increasing awareness of the condition in recent years. However, there is no general agreement on how to manage and treat this syndrome. This review looks at all published cases of 3M syndrome to understand how patients have been treated and how they responded to different therapies. By gathering this information, we aim to help doctors recognise 3M syndrome early and make informed treatment decisions that improve outcomes.

## 1. Introduction

Triple M (3M) syndrome is a severe immune-related complication of immune checkpoint inhibitor (ICI) therapy, characterised by the co-occurrence of myasthenia gravis, myositis and myocarditis. While each of these conditions may be triggered independently in the context of immunotherapy, the recognition of 3M syndrome as a distinct overlap syndrome is increasing, with reported mortality rates reaching 38% [1,2,3]. However, there is no consensus on the management of this condition, resulting in varied practice across different hospitals.

The individual components of this overlap syndrome are well recognised in the ICI-toxicity literature and international guidelines [4]. Among ICI-induced neuromuscular complications, myositis is the most prevalent [5], followed by peripheral neuropathies and then myasthenic syndromes [6]. Myocarditis, although less common, is a potentially life-threatening complication, and its simultaneous occurrence with neuromuscular disease presents significant challenges for diagnosis and management [7,8]. A report of 1726 cases of ICI-induced myositis and myasthenia indicated that mortality was higher in those with 3M (at 43.8%) compared to those with two out of any three of myocarditis and myositis or myasthenia gravis (at 29.8%) [9].

Recognition of 3M syndrome has improved, and this is reflected in an increasing incidence of descriptive case reports and small case series in the oncology, neurology and cardiology literature [1,2,9,10,11,12,13]. The syndrome has been incorporated into recent iterations of European Society of Medical Oncology (ESMO) ICI toxicity guidelines [4]. This guidance is directed at oncologists and relies on the Common Terminology Criteria for Adverse Events (CTCAE) grading to dictate therapeutic strategy. Corticosteroids are recommended as first-line therapy, with intravenous immunoglobulin (IVIg) or plasma exchange (PLEX) considered a second-line treatment for steroid-refractory cases. However, there are no standardised recommendations for steroid dosing and duration, third-line immunosuppression, or clear information on monitoring protocols, intervention thresholds or treatment escalation strategies. Managing these syndromes is complex and requires multi-speciality collaboration, as clinicians with expertise in the management of each individual element must balance monitoring and treatment priorities, alongside consideration of the underlying cancer and individual patient frailty, to optimise patient outcomes.

The purpose of this review is to examine reported cases of 3M syndrome in the context of ICI therapy, with a focus on treatment strategies, disease trajectory and patient outcomes. Previous reviews have discussed the demographic and clinical characteristics of patients who develop 3M and which ICI therapies are most associated with the condition (for review, please see Pathak et al. [2] or Lipe et al. [14]). This review will instead focus on the reported treatment strategies and patient outcomes. By synthesising the existing evidence, we hope to provide insights that will support clinicians in managing this complex condition and contribute to the development of future consensus guidelines.

## 2. Materials and Methods

Whilst this is a narrative review to enhance the quality of the included data, this study was conducted following a predefined protocol and adhered to the Preferred Reporting Items for Systematic Reviews and Meta-Analyses (PRISMA) guidelines (see Supplementary Material). The review was registered on the PROSPERO database [15] (registration number: CRD42024625987).

A comprehensive literature search was performed on PubMed, EMBASE and Jstor on 12 January 2024, with an additional search conducted on 20 December 2024. The search terms used are detailed in Table 1, and an additional full-text and bibliography search was undertaken to ensure completeness.

After removing duplicates, studies were screened for inclusion based on the following criteria: full-text, peer-reviewed articles published in English since 2010, reports including the combination of myasthenia gravis, myocarditis and myositis (occurring simultaneously), studies involving human subjects receiving ICI therapy in an oncology setting. Exclusion criteria included: studies focusing on animal or molecular models, reports where individual patient-level data were unavailable, and cases where immunotherapy was used outside of oncology. There were several studies that reported on only one component of 3M or two overlapping elements (such as myositis and myocarditis), but papers were not included unless all three conditions of the triad were present.

For studies that met initial inclusion criteria but lacked individual patient-level data, we contacted the corresponding authors to request additional information.

Data of interest were predefined and are outlined in Table 2. Two independent reviewers (I.W. and M.F.) extracted data using a standardised electronic data collection form. Any discrepancies were resolved through discussion with a third reviewer (A.C.). Diagnoses of 3M syndrome and its components were accepted as reported, and no retrospective reassessment was performed. Data pulled from the papers was that felt to be of interest to clinicians, including the screening and diagnostic tests used on presentation to hospital; the first-, second- and third-line treatments; and any further immunotherapy. We have also explored the patient outcomes and the risk of 3M relapse.

All statistical analyses were conducted using R (R version 4.3.3 (29 February 2024)). Due to variability in data availability across studies, not all cases were included in every analysis, and we were unable to perform a meta-analysis on the different treatment options. No ethical approval was required, as this study exclusively examined previously published data.

## 3. Results

A large amount of data is available within each case, and we therefore subclassified our results into different sections that may be of clinical interest, including a brief discussion of paper characteristics, patient presentation and screening tests. We then reviewed the first- and second-line treatments in greater detail, any further immunotherapies utilised, 3M syndrome relapse and eventual patient outcome. Due to the heterogeneity in reporting across studies, the number of patients included in each section varies, as not all studies report on each aspect consistently.

### 3.1. Paper Characteristics

**Figure 1:**
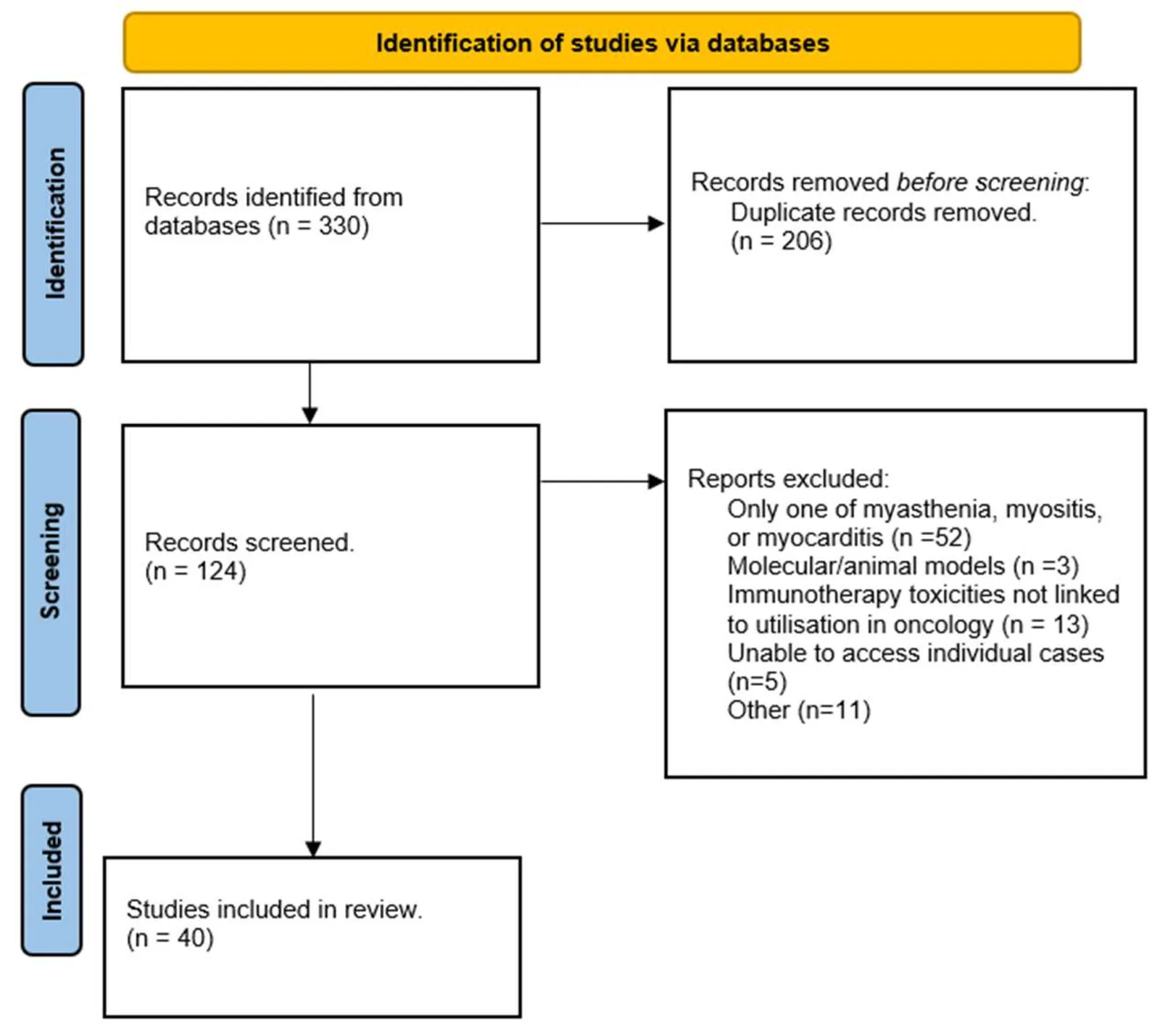
PRISMA 2020 flow diagram of identified studies [16].

The initial literature search identified 330 papers, with 247 retrieved through title and abstract screening and an additional 83 identified through full-text review and bibliography searches (Figure 1). After removal of duplicates, 124 articles were screened, and 40 papers were included in the final analysis.

The majority of the included studies were single case reports, although some case series and narrative literature reviews were also analysed. Several studies were excluded after full-text screening due to insufficient patient-level data, preventing a clear correlation between treatment, patient outcomes and disease progression. The total number of cases with sufficient detail for extraction is reported below. The numbers of cases and the variability of reporting limits us from performing a full systematic review with meta-analysis, hence subsequent results reported are in a narrative form.

### 3.2. Patient Presentation and Initial Screening

A total of 82 individual cases were identified, each providing treatment and outcome data at either a group or individual level. Patient characteristics are summarised in Table 3.

A range of ICIs were implicated in 3M syndrome, with pembrolizumab being the most common association (*n* = 29, Supplementary Figure S1). Among the 63 cases with sufficient clinical detail, 48 patients (76.2%) underwent screening for all three components of the syndrome upon hospital admission. However, many did not present with clear, overt features of all three overlapping conditions.

### 3.3. Diagnostic Assessments

**Figure 2:**
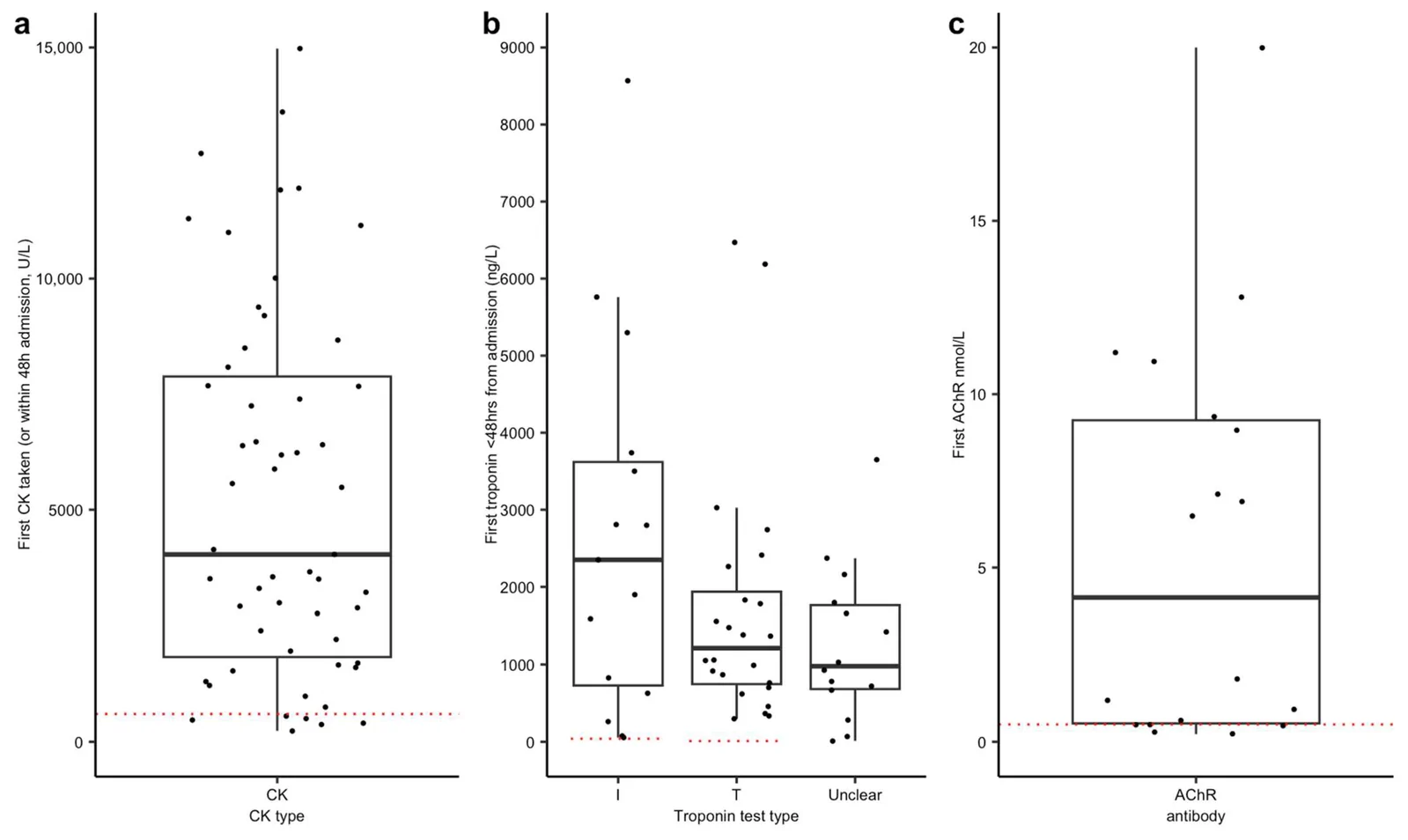
Box plots showing screening blood tests completed in the first 48 h from admission. Each dot represents a patient. (**a**) Tested CK results; a single CK-MB result has been excluded, two extreme outliers (values = 61,233 and 25,200) removed, displayed *n* = 56. (**b**) Troponin results. In both graphs, 4 extreme outliers have been excluded (values troponin I = 25,010 and 19,644; unclear = 36,000 and 16,450), displayed *n* = 53. (**c**) Reported AChR antibody concentrations (nmol/L) *n* = 18. Not all AChR results were reported numerically; there were an additional 9 cases with AChR results reported as positive and 37 reported as negative. The red dotted lines signify the upper limit of the normal ranges (**a**) CK 600 U/L, (**b**) troponin I = 40 ng/L, troponin T = 10 ng/L and (**c**) AChR 0.5 nmol/L (although reported normal values varied between reporting institutions).

Myositis: Creatine kinase (CK) or CK subtypes were the most frequently ordered test, performed in 58 (85.3% of 68 cases) cases within the first 48 h (Figure 2a), and were abnormal in 89.7% (for consistency across cases, abnormal was considered >600 U/L). However, a normal CK does not exclude the presence of immunotherapy-related myositis. EMG/NCS were completed in 17 cases (20.7%) and were reported as showing evidence of myositis in 12/17 studies (70.5% in whom they were performed). Full results are reported in Supplementary Table S1. Muscle biopsy was reported in ten cases, and MRI in five cases.

Myocarditis: Troponin (I or T) was measured within 48 h in 57 (83.8% of 68 cases) of cases (Figure 2b), with all cases reporting troponin T or I showing elevated levels. However, inconsistent reporting and timing of serial troponin measurements made it difficult to track disease progression. The majority of patients who underwent echocardiography had normal results, although many showed abnormalities on ECG. Baseline, pre-immunotherapy ECGs were not available for comparison in most cases. Initial admission ECGs were available in 40 cases (Table 4) and were normal in 13 (28.3%). Some initial ECGs showed more than one abnormality; we found evidence of conduction abnormality in 23 cases (57.5%), ischaemia in 6 cases (15%) and non-specific ST segment or T wave changes in 4 cases (10%). The endomyocardial biopsy result was reported in eight cases.

Myasthenia gravis: Diagnosis was based primarily on clinical assessment, with no consistent use of standardised scoring systems. This reflects the time taken to organise neurophysiological investigation, which is also difficult to perform in an ITU setting, as well as due to the turnaround time for antibody testing. AChR antibody results were detailed in 64 cases; a positive result (determined by reporting institution threshold values) was reported in 27 (42.2%) cases, with 18 cases reporting AChR titre (Figure 2c), and a negative result was reported in 37 cases. Other antibodies were less consistently reported; however, anti-MUSK antibodies were reported in 1 case, and anti-striated muscle antibodies were positive in 13 cases (anti-titin *n* = 5, anti-ryanodine *n* = 1). Anti-Kv1.4 was positive in three cases. Results of repetitive nerve stimulation were reported in 16 cases (19.5%), with abnormal decrement found in 5 cases (31.3% in whom they were performed).

### 3.4. First-Line Treatment

All patients received steroids as first-line treatment, but there was significant variation in the formulation, dose and duration. Methylprednisolone was the most commonly prescribed, followed by dexamethasone and prednisolone.

**Figure 3:**
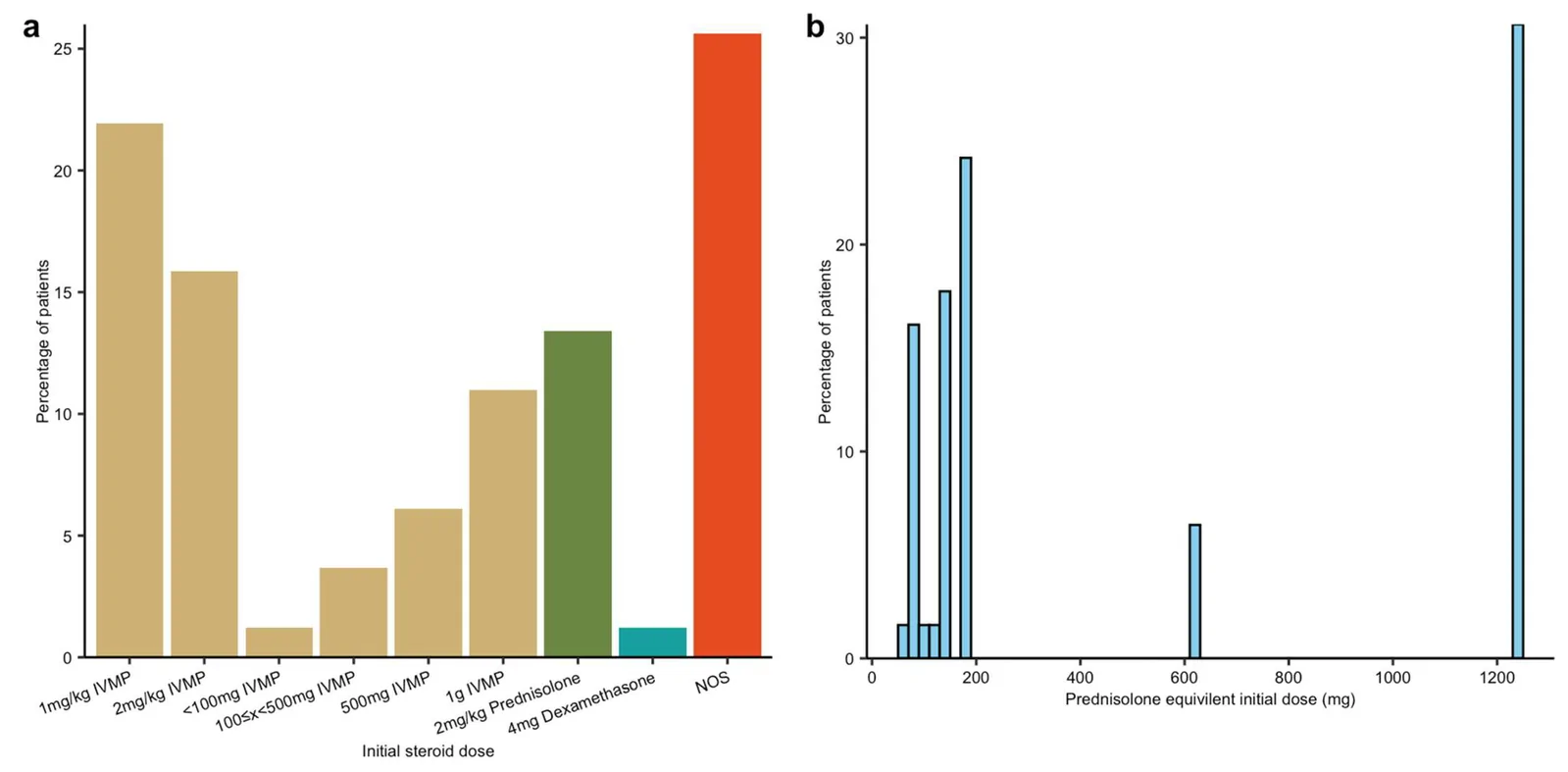
Bar graphs illustrating steroid use across included cases. (**a**) shows the percentage of patients receiving each type of steroid grouped by dosage ranges, and (**b**) shows the prednisolone equivalent dose for the initial steroid treatment of each patient to allow different steroid types to be compared, shown as a percentage of patients. For dosage ranges, the middle of the range was taken. For patients for whom the dose was given per weight, we assumed a weight of 70 kg to calculate a dose in mg. NOS = not otherwise specified.

To facilitate comparison, we converted all initial steroid doses to a prednisolone-equivalent dose. For cases where dose ranges or weight-based dosing were provided, we assumed a 70 kg body weight and used the midpoint of the reported range. Initial steroid doses ranged from 75 mg/day to 1250 mg/day (Figure 3). Usage was broadly grouped into a low-dose (equivalent to 1–2 mg/kg IV methylprednisolone) and high-dose range (equivalent to 500–1000 mg IV methylprednisolone), which corresponds to 2022 ESMO guidelines for the treatment of the 3M component which was viewed to be the predominant disease manifestation [4].

The duration of steroid administration also varied. Many patients received high-dose methylprednisolone pulses initially, converting to oral prednisolone during the acute phase of illness. There was no available data on whether patients received steroid prophylaxis, such as bone protection or gastric protection. The reason for steroid duration/cessation was not consistently documented.

Acetylcholinesterase inhibitors (AChRIs) were prescribed for 37 patients, with pyridostigmine (*n* = 34) being the most used, followed by neostigmine (*n* = 4), with one patient receiving both. Given the myocarditis-induced conduction abnormalities in 3M syndrome, AChRIs pose a potential risk for cardiac dysfunction, as they are known to cause bradycardia and hypotension [17]. To assess the severity of cardiac involvement, we used the requirement for temporary cardiac pacing or pacemaker implantation as an indicator of severe cardiac dysfunction. While 12 patients required a pacemaker, there was a causative link between the timings of AChRI treatment and pacing that could not be established. Three patients had new conduction deficits noted on ECG after AChR administration (including atrial fibrillation, complete heart block and QT prolongation); however, again, the timing of these events was unclear.

### 3.5. Second- and Third-Line Treatments

Following steroids, intravenous immunoglobulin (IVIg) and plasma exchange (PLEX) were the most used treatments. Overall, 54 patients received IVIg, 27 received PLEX and 12 patients received both. Among those who received both therapies, PLEX was administered first or at a >4-week interval after IVIg in four cases. In four additional cases, the treatment sequence could not be determined. However, in the remaining four cases, PLEX was performed shortly after IVIg, with the potential to negatively impact on immunoglobulin mechanism of action.

Among the 47 cases with sufficient clinical detail, 27 (57.4%) experienced worsening of myasthenia symptoms after steroid treatment, with 14 requiring ventilatory support (66.6% of 21 cases with sufficient description). To assess whether early immunomodulatory therapy could prevent post-steroid deterioration, we analysed the timing of PLEX or IVIg initiation relative to steroid administration. Early immunotherapy was defined as PLEX or IVIg given ≤10 days before or after steroid initiation. Among patients whose myasthenic symptoms worsened post-steroids, 25.9% received late or no immunomodulatory therapy (55.56% early or immunotherapy first, 18.52% unclear timing, total *n* = 27), compared to 10% in those who did not worsen (60% early or immunotherapy first, 30% unclear timing, total *n* = 20). The post-steroid trajectory was unclear in 35 cases. Case numbers were too small, and outcome reporting was not granular enough for definitive conclusions to be made.

### 3.6. Further Immunotherapy

**Figure 4:**
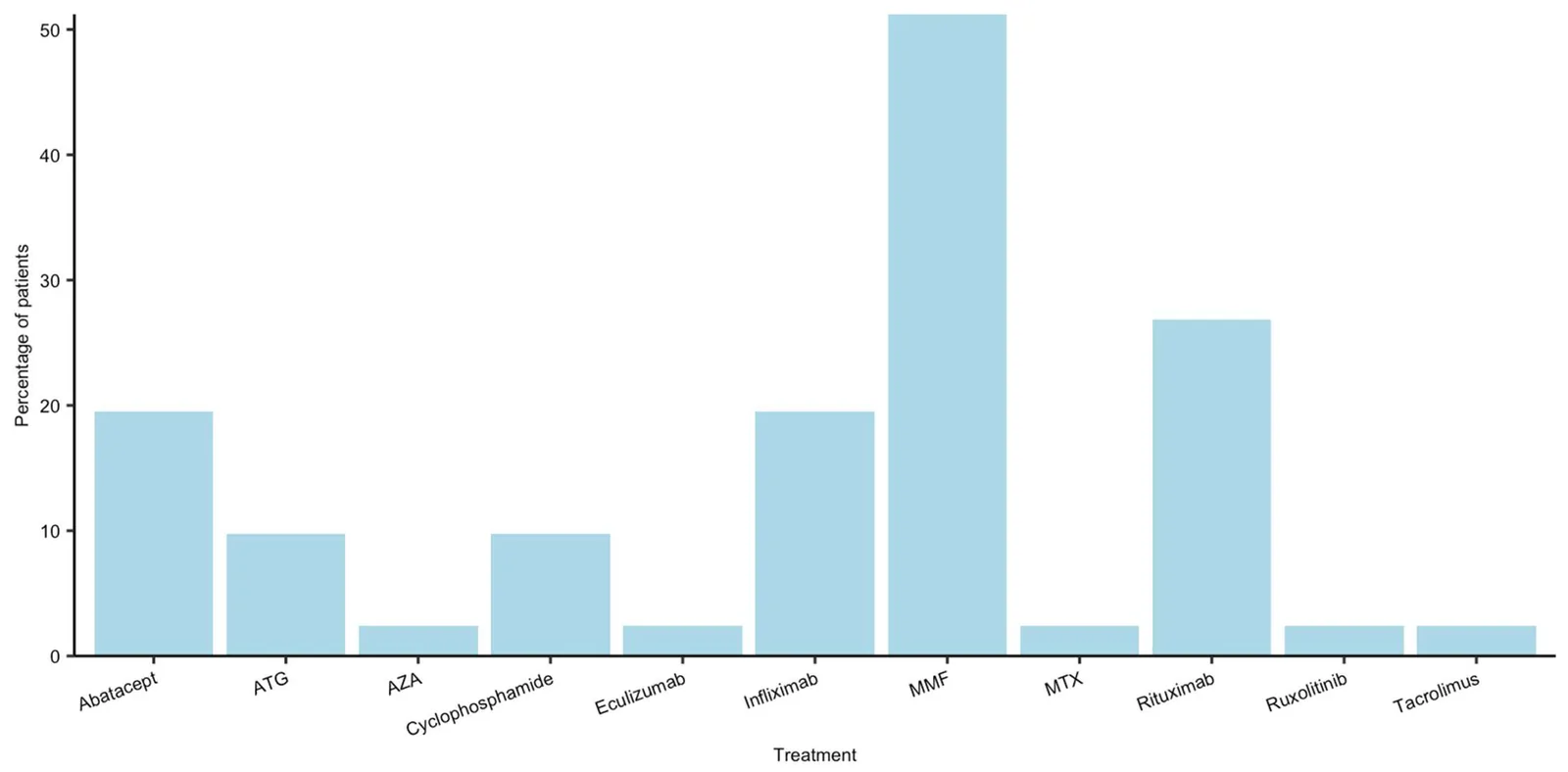
Choices of second-line treatment were detailed in 41 cases. Overall, 61 second-line treatments were used, with some patients receiving more than one therapy. Shown is of percentage of patients who received second-line therapy (*n* = 41) for each drug.

Further immunotherapeutic options were reported in 41 cases, often in combination. There was limited discussion regarding agent selection or whether the choice was targeted to the treatment of refractory myocarditis or refractory myasthenia primarily. The timing of the introduction of the new agent was not consistently discussed in the body of the reports. Twelve patients received two additional immunosuppressive agents (beyond steroids, PLEX and IVIg). Four patients received three additional agents. The most frequently used second-line treatments were Mycophenolate (*n* = 21/51.2%), Rituximab (*n* = 11/26.8%), Infliximab (*n* = 8/19.5%) and Abatacept (*n* = 8/19.5%). This is illustrated in Figure 4.

In several cases, some symptoms recurred during steroid tapering within the initial admission. The duration of admission was not consistently reported.

### 3.7. Patient Outcomes

3M syndrome was associated with a high mortality rate, with 37.8% of patients succumbing to the condition. Among cases with sufficient clinical detail, 41% (*n* = 34) died from ventilatory failure (*n* = 14).

Beyond mortality, there was significant morbidity among affected patients, though CTCAE grading was inconsistently reported. Similarly, ECOG performance status before and after admission was not consistently documented. We are therefore unable to comment on precise endpoints for this condition other than mortality.

Of the 54 cases with sufficient detail, 36 patients (66.7%) required admission to the intensive care unit (ICU). Where detailed (*n* = 34), the most common reason for ICU escalation was respiratory failure requiring ventilatory support (73.5%, *n* = 25). Among 68 cases with available respiratory support data, 34 patients required respiratory support, and some patients received multiple modalities; 24 required mechanical ventilation, and 13 required non-invasive ventilation (NIV).

Variation in troponin assays and differences, frequency and timing of measurement prevented analysis of troponin trends. A variety of cardiac arrhythmias were noted, with 17 patients requiring cardiac pacing, with permanent pacemaker insertion in 13 patients.

### 3.8. 3M Relapse

An important consideration in 3M syndrome is the risk of relapse. However, follow-up duration was often limited, with many reports not extending beyond hospital discharge.

With this limitation, myasthenia relapse was reported in three cases. The first is included as part of a group analysis and states a relapse of myasthenia occurred within 7 months (ref case 11). The second patient experienced a worsening of their myasthenic symptoms whilst on azathioprine maintenance therapy after steroids had been weaned; however, this patient opted for ICI rechallenge due to cancer remission (ref case 19). The final patient had been discharged to a rehabilitation facility after receiving methylprednisolone and IVIG and re-presented to the hospital a week later with features of a myasthenic crisis. There were several additional cases where 3M symptoms reoccurred with steroid weaning during the index admission, which we have not classed as relapses and likely represent the clinical course of a monophasic presentation.

## 4. Discussion

This narrative review highlights 3M syndrome as an emerging and serious immune-related complication of cancer immunotherapy with a high mortality rate and frequent need for intensive care support. In our review we have reported on the different treatments utilised; however, treatment variability and inconsistency of clinical monitoring makes it difficult to draw broad conclusions about the most effective treatment strategies, Additionally, it is unclear if different treatments may be more efficacious depending on the ICI agent, or whether other patient characteristics should guide treatment choice. There is current work to develop a 3 M-specific clinical practice guideline, which, with multi-speciality input, may inform rational clinical monitoring, introduce treatment thresholds, guide management and potentially improve patient outcomes.

As ICI use continues to expand, it is critical to increase awareness of 3M, particularly amongst Emergency Department, Acute Medicine, Acute Oncology, Neurology, Rheumatology and Cardiology services. In our review, we found that most patients had appropriate and early screening. However, the utilisation of different screening tests remains an area of debate. For example, we saw that CK was a commonly utilised test; however, it has been noted that this test can be negative in ICI-associated myositis [18]. Similarly, with myocarditis, Troponin-I can be negative on screening (although Troponin-T is more frequently positive), again indicating that a negative test does not entirely rule out this condition [19]. However, these tests provide valuable information quickly in the acute setting and, when abnormal, can provide early evidence to suggest skeletal or cardiac muscle involvement, prompting more conclusive tests whilst acknowledging their limitations. Given the high mortality of 3M syndrome, early recognition is critical to facilitate time-sensitive multi-speciality discussions and allow appropriate escalation of clinical support. Provisional guidelines for the screening, classification and early treatment of 3M, including the use of steroids, have been published. However, given the complexity of this condition, further work involving multi-speciality input is likely needed [20]. The significant variability in steroid dosing, route of administration (oral vs. intravenous) and duration of therapy provides strong support for this suggestion. It may be that the utilisation of different doses may reflect differences in ESMO management guidelines [4] for the individual components that make up 3M syndrome and their perceived severity grading; however, this was difficult to ascertain from the studies. From the ICI-neurotoxicity literature, disease refractory to steroid treatment is a key concern [21,22]. However, it was difficult to determine the presence of steroid failure as new treatments were often added quickly without accompanying justification or the reporting of further tests. Whilst we observed that all patients had steroids, the reporting of steroid safety interventions, including bone and gastrointestinal protection and use of anti-microbial prophylaxis, is poor. A reported complication of isolated high-dose steroid treatment in myasthenia gravis is the “myasthenic dip”, a transient worsening of symptoms with 10 days of steroid initiation [23,24]. While neurologists are familiar with this phenomenon and either introduce steroid gradually or treat myasthenic crisis with contiguous intravenous immunoglobulin or PLEX, awareness among other specialities may be more limited. Although we found evidence of a deterioration of myasthenia symptoms in 56% of patients (27 of 48 cases) following steroid administration, with 66% (14 of 21 cases) requiring ventilatory support, it remains unclear whether this deterioration was directly caused by steroids or if it reflected the natural disease progression. However, if we take direction from decades of experience in the management of analogous autoimmune conditions, as is recommended in the 2022 ESMO guidelines, we should acknowledge the potential for isolated corticosteroid treatment to exacerbate the myasthenic element of 3M syndrome.

Second-line immunotherapy choices varied widely amongst cases, and the rationale behind choosing different therapies was not explored in each paper. There is also an inconsistency in clinical monitoring, which makes it difficult to interpret the therapeutic impact of individual agents. The variation in the choice of second-line therapies may reflect a combination of factors, including the local experience of the treating team, local drug availability, speed of treatment onset and the dominant condition amongst the 3M triad in the individual case. Choice of second-line therapy, and immunosuppression in general, requires balanced consideration of the risk from the ir-AE and potential impact on cancer outcome. While a detailed discussion is beyond the scope of this review, a variety of treatment considerations exist. Efforts can be made to target the underlying pathological process based on currently available evidence. Myasthenia gravis is the archetypal antibody-mediated autoimmune disease, driven and is B cells driven and hence, where myasthenia is the primary concern, treatments such as rituximab are often preferred [25]. In ICI-myositis, there is evidence of upregulation of the IL-6 pathway, and hence, treatments such as tocilizumab may be favoured [26]. Ongoing research into myocarditis has targeted the same pathway but with JAK2 inhibitors and/or abatacept [27,28].

These decisions are further complicated by the need to preserve a therapeutic oncological effect from ICI therapy. For instance, abatacept, which blocks the interaction between T cells and antigen-presenting cells, broadly impairing the T-cell response and reversing pathways activated by ICI therapy, may therefore adversely impact cancer outcome. Given the complexity of these treatment choices and the necessity to balance competing treatment goals, decisions should involve multi-speciality discussion and may benefit from input provided by specialist centres. This is especially important as the mechanisms underlying 3M syndrome remain poorly understood.

Interestingly, this review suggests that 3M syndrome generally follows a monophasic disease course, with minimal recurrence after initial treatment. However, few studies provided long-term follow-up data, limiting our ability to assess the true risk of late recurrence. But the potential for exacerbation with ICI-rechallenge should be part of the discussion when considering further oncological treatments. Currently, there is limited data on the recurrence of complications on rechallenge, and caution has been advised [4,29]. Although ongoing monitoring was not a major focus in the reviewed studies, late relapses of ir-AEs have been reported [30,31]. However, it remains unclear which specific complications are more likely to recur.

Whilst our paper explored a large number of individual 3M cases and provided a depth review of their treatments, there were several limitations. Reported case studies are often likely to be cases felt to be more interesting to the broader scientific audience, meaning that they may be more unusual cases or those with an interesting trajectory. However, although there is likely to be publication bias skewed towards more severe or unusual presentations, the authors‘ clinical experience reflects the severity reported here. We also had strict inclusion criteria, meaning that some interesting studies were not included, in particular those focused on a dual overlap rather than triad overlap syndrome. We are also aware that additional studies have been published since we began our analysis, and these may offer valuable contributions to the broader literature. The increasing frequency of the publication of these papers in recent years further emphasises the importance of understanding the available treatment options and their effects [32,33,34,35].

This study did not aim to develop a formal treatment guideline, but rather to provide an overview of the various treatment approaches reported. The available data illustrate a wide variation in practice and a lack of cohesive treatment strategies, which, alongside poor reporting of individual outcomes, severely limits our ability to provide specific treatment recommendations and any future recommendations would likely need to be consensus-based. Given the small number of cases and the limited detail on treatment regimens and morbidity, conducting meaningful comparative analysis is currently not feasible. We did attempt to perform an analysis of steroid dosing or the timing of second-line therapy introduction; however, the limited information on treatment timings made this too difficult. Any such effort would risk overinterpretation of highly fragmented data.

Moreover, key outcome measures, such as morbidity and cause of death, were often inconsistently reported, highlighting an important area for improvement in future research. The absence of standardised diagnostic criteria, inconsistent descriptions of clinical features and the diversity of treatment regimens all hinder the ability to draw firm conclusions or offer evidence-based recommendations. Within the limitations of the available evidence, our recommendations can only reflect broad clinical themes rather than prescriptive guidelines. These include the use of screening tests for all suspected cases, even in individuals presenting with only a single component of the clinical triad; early involvement of intensive care, given the high morbidity and mortality; and timely consultation with relevant specialities. With regard to specific treatments, early administration of high-dose steroids combined with PLEX or IVIG appears warranted based on current reports. It remains difficult to determine an optimal subsequent agent, but we suggest this should be guided by local availability and institutional familiarity. As further data emerge, we hope more specific and evidence-based treatment guidance will evolve.

Moving forward, there is a clear need for collaborative efforts to establish consensus definitions and standardised outcome measures. Prospective data collection and the creation of structured registries would significantly improve our understanding of disease patterns, therapeutic responses and long-term outcomes. In parallel, the development of a treatment algorithm, currently in progress, represents a critical step toward supporting clinicians in the management of this complex and evolving syndrome.

## 5. Conclusions

This is the first review focused specifically on the treatment approach to 3M syndrome. Due to the heterogeneity of included studies, we were unable to perform a meta-analysis, but by incorporating data from diverse settings and geographic locations, we hope our findings are widely generalisable. Here we highlight the complexity of diagnosing and managing this condition in the absence of strong clinical evidence to support monitoring and treatment strategy. Our findings offer practical insights for clinicians and underline the importance of developing consensus-driven treatment recommendations for this severe and emerging clinical entity.

## Figures and Tables

**Table 1:** Search terms used to identify relevant peer-reviewed literature.

	Search Terms
1	“Triple M” OR “Triple M Syndrome”
2	“Triad Overlap” OR “Triad Overlap Syndrome”
3	“Immune checkpoint inhibitors” OR “Checkpoint inhibitor” OR “Immunotherapy “ OR “PD-1 Inhibitor” OR “PD-L1 Inhibitor” OR “Anti-PD-1” OR “Anti-PD-L1” OR “CTLA-4 Inhibitor” OR “Anti-CTLA4”
4	“Myasthenia Gravis” OR “Myasthenia” AND “Myositis” AND “Myocarditis”
5	1 AND 3
6	2 AND 3
7	3 AND 4

**Table 2:** Details of the information extracted. AChEI = Acetylcholinesterase inhibitor, ICI = immune checkpoint inhibitor, ICU = intensive treatment unit, CK = creatine kinase.

Patient Details	Patient Age, Sex Cancer Type, Cancer Stage Pre-ICI Performance Status
Phenotype	Presenting symptoms
Immunotherapy	ICI type, time between ICI and presentation Steroid treatment: dose, regimen, days of IVMP, prednisolone equivalent dose Second line treatment: IVIg/PLEX/both treatments, time between initiation of steroids and initiation of second line therapy Third line treatment: treatment type, treatment dose and regime, indication for treatment escalation
Intensive care treatment	ICU admission, reason for ICU admission Ventilation requirement, ventilation method
Myocarditis	Initial troponin (T/I) taken within 48 h of admission, echocardiogram performed, early vs. late echocardiogram (defined as ≤10 days from admission) and echocardiogram findings Cardiac dysfunction prior to AChEI, cardiac dysfunction post AChEI Pacing requirement (temporary/permanent), pacemaker insertion before/after AChEI
Myasthenia Gravis	AChEI treatment Evidence of myasthenia worsening post steroid treatment, days between steroid treatment and myasthenia worsening Evidence of myasthenia relapse
Myositis	First CK taken within 48 h
Outcome	Mortality, cause of death Immunotherapy discontinuation

**Table 3:** Patient characteristics.

Age (Median ± IQR)	72 ± 11
% Female	34.9%
Cancer type:	
Malignant Melanoma	24 (34.8%)
Renal cancer	12 (17.4%)
Lung cancer	11 (15.9%)
Other	22 (31.9%)

**Table 4:** Conduction abnormalities detected on admission ECGs and subsequent conduction abnormalities that developed during admission. Forty cases provided admission ECGs; percentages are based on this value, and some cases developed more than one abnormality.

	Conduction Abnormalities at Presentation		Subsequent Conduction Abnormalities During Admission	
	*n*	% of Patients	*n*	% of Patients
No abnormality	17	42.5	28	70.0
RBBB	13	32.5	0	0.0
LBBB	2	5.0	2	5.0
Bundle branch block NOS	2	5.0	0	0.0
Bifasicular block	1	2.5	0	0.0
Trifasicular block	1	2.5	2	5.0
First-degree AV block	1	2.5	0	0.0
Second-degree heart block	0	0.0	1	2.5
Complete heart block	4	10.0	10	25.0
High-grade AV block	0	0.0	1	2.5
AV block NOS	1	2.5	2	5.0
QT prolongation	1	2.5	1	2.5

## Data Availability

Data is available upon request.

## Supplementary Materials

The following supporting information can be downloaded at: https://www.mdpi.com/article/10.3390/cancers17132063/s1, Table S1: Neurophysiology Results, Figure S1: Bar graph illustrating the immunotherapies implicated in the development of 3M syndrome grouped by mechanism of action.
