# Correction: Furlepa et al. Management of Triple M Syndrome: A Systematic Review with Narrative Synthesis of Immune Checkpoint Inhibitor-Induced Myasthenia Gravis, Myositis and Myocarditis. *Cancers* 2025, *17*, 2063

**DOI:** 10.3390/cancers18152503

**Published:** 2026-08-05

**Authors:** Martin Furlepa, Isabella Watts, Aisling S. Carr

**Affiliations:** 1 Department of Neurology, University College Hospital, London NW1 2BU, UK; 2 Department of Oncology, Imperial College Healthcare NHS Trust, University College London Hospital, University College London, London W6 8RF, UK; 3 Centre for Neuromuscular Diseases, National Hospital for Neurology and Neurosurgery, UCLH, London WC1N 3BG, UK; 4 Department of Neuromuscular Diseases, Institute of Neurology UCL, London WC1N 3BG, UK

## Title Error

In the published publication [1], there was an error regarding the title of the manuscript. The correct title is as follows: Management of Triple M Syndrome: A Systematic Review with Narrative Synthesis of Immune Checkpoint Inhibitor-Induced Myasthenia Gravis, Myositis and Myocarditis. The authors apologize for any inconvenience caused and state that the scientific conclusions are unaffected. This correction was approved by the Academic Editor. The original publication has also been updated.
