# Supplementary material for: Management of Triple M Syndrome: A Narrative Review of Immune Checkpoint Inhibitor-Induced Myasthenia Gravis, Myositis and Myocarditis
Source: Cancers (Basel). 2025 Jun 20;17(13):2063. doi: 10.3390/cancers17132063 (PMC12249040; doi:10.3390/cancers17132063)
Supplement: Supplementary file 1 [file cancers-17-02063-s001.zip › cancers-3641542-supplementary.pdf]

Supplementary Table S1: Neurophysiology Results.

case_id	NCS Result	EMG	Single Fibre	RNS	NCS
19	Electroneuromyography studies performed in the polyneuropathy and myasthenia protocol were within normal limits.	Normal	Normal	Normal	Normal
21	No abnormality was found in electromyography	Normal	Unclear if performed	Unclear if performed	Unclear if performed
22	Paraclinical investigations (body computed tomography scan (body CT) and ENMG) discarded neurological diseases, specifically multiple sclerosis, MG, demyelinating polyradiculoneuropathy, and other peripheral neuropathies.	Normal	Normal	Normal	Normal
24	Paraclinical investigations (body computed tomography scan (body CT) and ENMG) discarded neurological diseases, specifically multiple sclerosis, MG, demyelinating polyradiculoneuropathy, and other peripheral neuropathies.	Normal	Normal	Normal	Normal
40	No neuromuscular junction dysfunction	Unclear if performed	Normal	Normal	Unclear if performed
41	Axonal and demyelinating motor and sensory peripheral neuropathy	Unclear if performed	Unclear if performed	Unclear if performed	Normal
42	Signs of myositis	Myositis	Unclear if performed	Unclear if performed	Unclear if performed
45	On electromyography, the examined muscles showed myogenic	Myositis	Unclear if performed	Unclear if performed	Unclear if performed

	damage, and the left deltoid showed a spontaneous spot (active phase) manifestation				
46	Electromyography showed muscle unit potentials with reduced amplitude and short duration, as well as increased fibrillation and positive sharp weave in her biceps brachii, extensor digitorum communis and quadriceps femoris. Repetitive stimulation of the facial, accessory and ulnar nerves did not reveal significant decrement or increment.	Myositis	Unclear if performed	Normal	Unclear if performed
49	Electromyography (EMG) revealed fibrillation potentials and positive sharp waves with normal recruitment and motor unit action potential morphology in keeping with acute inflammatory myopathy (IM); repetitive nerve stimulation was normal.	Myositis	Unclear if performed	Normal	Unclear if performed
50	An EMG revealed fibrillations, positive sharp waves, and early recruitment showing instability of the neuromuscular junction. Repetitive stimulation was normal.	Myositis	Unclear if performed	Normal	Unclear if performed
54	sensori-motor symmetrical polyneuropathy, with axonal and demyelinating features. Furthermore, a decremental response was detected at the	Myositis	Unclear if performed	Abnormal	Neuropathy

	repetitive 3 Hz stimulation, without post-exercise increase. The concentric needle electrode revealed a myopathic pattern at interference; spontaneous activity appeared two weeks later.				
55	sensori-motor symmetrical polyneuropathy, with axonal and demyelinating features. Furthermore, a decremental response was detected at the repetitive 3 Hz stimulation, without post-exercise increase. The concentric needle electrode revealed a myopathic pattern at interference; spontaneous activity appeared two weeks later.	Myositis	Unclear if performed	Abnormal	Neuropathy
56	Electrophysiologic neuromuscular junction tests, including edrophonium test and repetitive nerve stimulation test, were negative.	Normal	Normal	Normal	Normal
57	EMG demonstrated abnormal spontaneous activity in both gastrocnemius muscles	Myositis	Unclear if performed	Unclear if performed	Unclear if performed
58	Consistent with myopathy with associated muscle membrane irritability. Interpretation of 3 Hz repetitive nerve stimulation limited by movement artefact but showed decremental response in compound motor action potential	Myositis	Unclear if performed	Abnormal	Unclear if performed

	amplitude (up to −10.7%)				
68	Myopathic changes consistent with myositis	Myositis	Unclear if performed	Unclear if performed	Unclear if performed
72	no fibrillation potentials, pathological recruitment of the iliopsoas, a repetitive nerve stimulation test of the left facial nerve did not reveal a waning pattern.	Myositis	Unclear if performed	Normal	Unclear if performed
73	There were changes in keeping with myopathy on needle electromyography, but repetitive stimulation showed no decline in amplitude.	Myositis	Unclear if performed	Normal	Unclear if performed
79	Negative repatative nerve stimulation	Unclear if performed	Unclear if performed	Normal	Unclear if performed
86	Fatigable stimulation	Unclear if performed	Unclear if performed	Abnormal	Unclear if performed
87	Electrophysiological tests showed no waning of amplitude in repetitive stimulation tests of the orbicularis oculi and abductor pollicis longus.	Unclear if performed	Unclear if performed	Abnormal	Unclear if performed

Supplementary Figure S1.

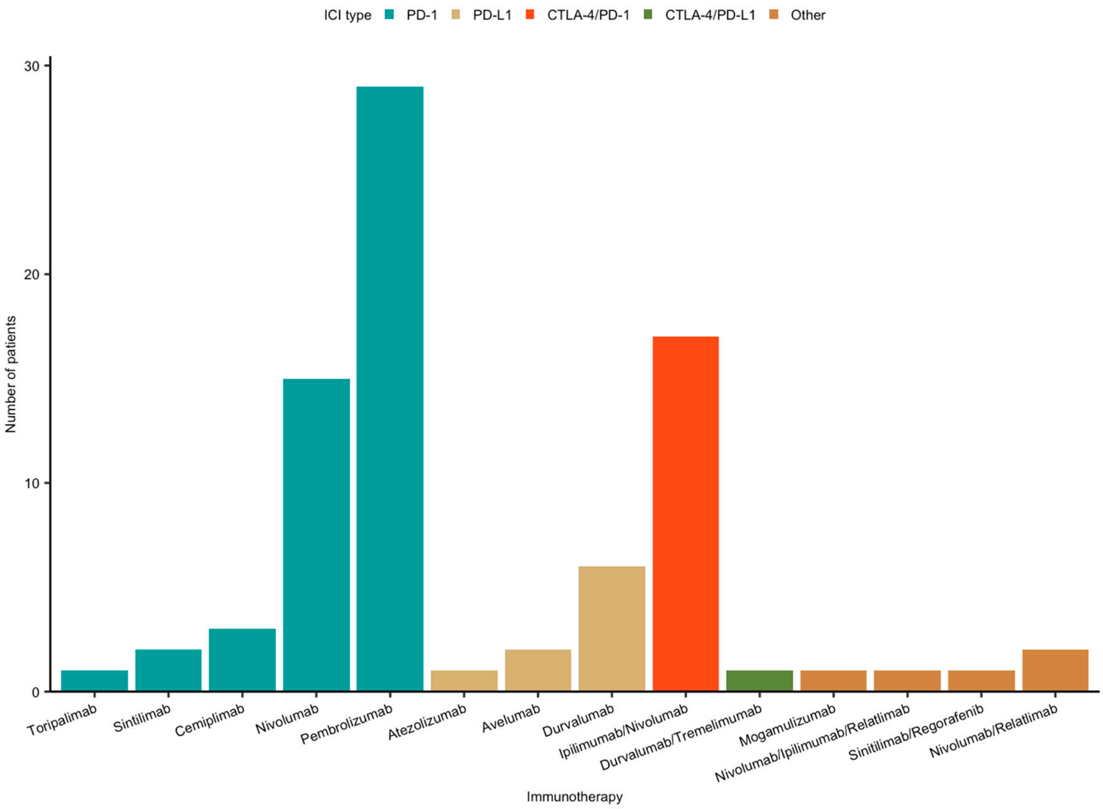

Figure S1 bar graph illustrating the immunotherapies implicated in the development of 3M syndrome grouped by mechanism of action.
